# Supplementary material for: End-to-end multimodal structure elucidation from raw spectra combining contrastive learning and evolutionary algorithms
Source: Nat Commun. 2026 Jun 5;17:5013. doi: 10.1038/s41467-026-73846-y (PMC13241512; doi:10.1038/s41467-026-73846-y)
Supplement: Supplementary file 1 — Supplementary Information [file 41467_2026_73846_MOESM1_ESM.pdf]

Supplementary Information

Supplementary Note A Technical advances

To create the first system that fulfills all desired characteristics in Fig. 2, we bring together several key technical innovations that address fundamental limitations in existing structure elucidation approaches:

- **Multimodal spectroscopic-molecular contrastive alignment:** We developed the first framework to contrastively align embeddings from heterogeneous spectroscopic modalities (^{1}H-NMR, ^{13}C-NMR, HSQC, IR) with molecular structure embeddings in a unified latent space. This required designing specialized encoder architectures for each spectroscopic modality (1D CNNs with self-attention for ^{1}H-NMR, 2D ResNet-style CNNs for HSQC, MLPs for ^{13}C-NMR) while ensuring cross-modal semantic alignment. This technical advance enables direct cross-modal retrieval between any spectroscopic measurement and molecular databases—dramatically expanding searchable chemical space beyond existing spectral databases.

- **Cross-modal retrieval-seeded evolutionary optimization:** We introduced a novel hybrid approach that combines cross-modal retrieval with discrete molecular optimization. Retrieved molecules from our aligned embedding space serve as initial populations for a genetic algorithm operating directly on molecular graphs. This addresses the critical cold-start problem in evolutionary molecular design by providing chemically-relevant starting points rather than random initialization, leading to faster convergence and higher-quality solutions.

- **Physics-informed spectral augmentation pipeline:** We developed a comprehensive augmentation framework that transforms idealized simulated spectra into realistic experimental-like data through physically-motivated perturbations including J-coupling simulation, phase errors, baseline drift, and instrumental noise modeling. This technical innovation enables models trained on simulated data to generalize to real experimental measurements.

- **Calibrated uncertainty quantification:** We established that cosine similarities in our aligned embedding space serve as well-calibrated confidence estimates, with empirical validation showing almost linear correlation between similarity scores and prediction accuracy. This enables reliable uncertainty quantification essential for practical deployment.

- **Systematic human-AI performance benchmarking:** We conducted the first controlled study comparing automated structure elucidation against expert

chemists on identical molecular identification tasks, establishing quantitative baselines for human-level performance in this domain. We compiled novel datasets to allow us to do so.

These technical advances synergistically address the five key limitations of existing approaches: requirement for manual preprocessing, inability to combine multiple spectroscopic modalities, lack of confidence estimates, restriction to database compounds, and absence of relevant contextual information for interpretation.

Supplementary Note A.1 Comparison to other works

We also compare SECS to past attempts to build CASE systems. In Supplementary Table 1, we delineate the differences, exposing some fundamental limitations of past approaches. In this table, 9 different criteria are evaluated, that can be placed in three distinct groups: overall system capabilities (dereplication, elucidation, end-to-end, calibrated uncertainty, adaptable chemical space), limitations (max heavy atom count, easy to add modalities) and evaluation (expert comparison, raw experiment evaluation). Our system stands out as the most robust approach for structure elucidation, encompassing all the attributes of a complete CASE system. On the evaluation side, this work is the only one that compares to NMR experts, providing us with an *approximate upper bound* for the human performance on a narrow dataset (9/20 molecules solved by the most experienced NMR expert with circa 20 years of experience).

Below we define the terms from Supplementary Table 1 one-by-one:

- **Dereplication** The ability of a CASE system to identify a molecule based on already collected samples.
- **Elucidation** The ability of a CASE system to propose new structures.
- **End-to-end** The ability of a CASE system to start directly from the raw data and end with proposing the final candidates without manual inputs / interventions such as lists of picked peaks.
- **Calibrated uncertainty** The ability of the system to provide confidence estimates for its own predictions.
- **Adaptable chemical space** The ability of the system to adapt its proposals depending on an input database.
- **Max heavy atoms** This represents a limitation denoting the maximum number of heavy atoms in a target molecule.
- **Easy to add modalities** The ability of the system to easily incorporate new types of spectra without retraining any of the existing models.

- **Expert comparison** The existence of a comparison to expert spectroscopists.
- **Raw experiment evaluation** The system should be evaluated on raw (realistic) experimental data, and not idealized spectroscopic analysis with only a solvent and the target molecule.

Supplementary Table 1: Comparing the technical capabilities of SECS with prior works on structure elucidation. We define the fundamental properties of a complete CASE system and compare SECS to other approaches using these properties as criteria. HA = Heavy Atoms.

	Overall System Capabilities					Limitations		Evaluation	
**System**	**Dereplication**	**Elucidation**	**End-to-end**	**Calibrated uncertainty**	**Adaptable chem. space**	**Max HA count in development**	**Easy to add modalities**	**Expert comparison**	**Raw experiments evaluation**
**SECS** (This work)	✓	✓	✓	✓	✓	35	✓	✓	✓
Structure confirmation									
DP4-AI[1]	×	×	×	×	×	N/A	×	×	✓
Partial structure identification									
Enders et al.[2]	×	×	×	×	×	N/A	×	×	✓
Lee et al.[3]	×	×	×	×	×	N/A	×	×	×
Alberts et al.[4]	×	×	×	×	×	35	×	×	×
Unimodal elucidation									
Sridharan et al.[5]	×	✓	×	×	×	10	×	×	×
Alberts et al. (2025)[6]	×	✓	×	×	×	35	×	×	✓
Alberts et al. (2024)[7]	×	✓	×	×	×	35	×	×	✓
Kanakala et al.[8]	✓	✓	×	×	×	9	×	×	×
Wu et al.[9]	×	✓	×	×	×	13	×	×	×
Multimodal elucidation									
Alberts et al. (2023)[10]	×	✓	×	×	×	35	×	×	×
Pesek et al.[11]	×	✓	×	×	×	19	×	×	×
DeepSPInN[12]	×	✓	✓	×	×	10	×	×	×
Sherlock[13]	✓	✓	×	×	×	40	×	×	×
Hu et al.[14]	×	✓	×	×	×	19	×	×	×
MultiModalTransformer[15]	×	✓	×	×	×	35 (500 Da)	×	×	×

Supplementary Note B Dataset overview

All datasets are available on HuggingFace.

Supplementary Table 2: An overview of all the used datasets in this study.

Dataset	Modalities	Count	Source
Simulated dataset (training)	^{1}H NMR, ^{13}C NMR, IR, HSQC	705k	Alberts *et al.*[16]
Simulated dataset (test)	^{1}H NMR, ^{13}C NMR, IR, HSQC	38.8k	Alberts *et al.*[16]
Multimodal experimental dataset for finetuning	^{1}H NMR, ^{13}C NMR	2,370 ^{1}H NMR, 19,654 ^{13}C NMR	in-house + NMRshiftDB
Multimodal experimental dataset for testing	^{1}H NMR, ^{13}C NMR	34 each	in-house + NMRshiftDB
Chemotion dataset	^{1}H NMR	1.49k	mined from Chemotion repository

Supplementary Note C SECS workflow

SECS utilizes a novel “retrieve, then refine” approach to flexibly incorporate inductive biases (via the reference database) but also allow for scalability. Supplementary Fig. 1 gives an overview of the SECS workflow.

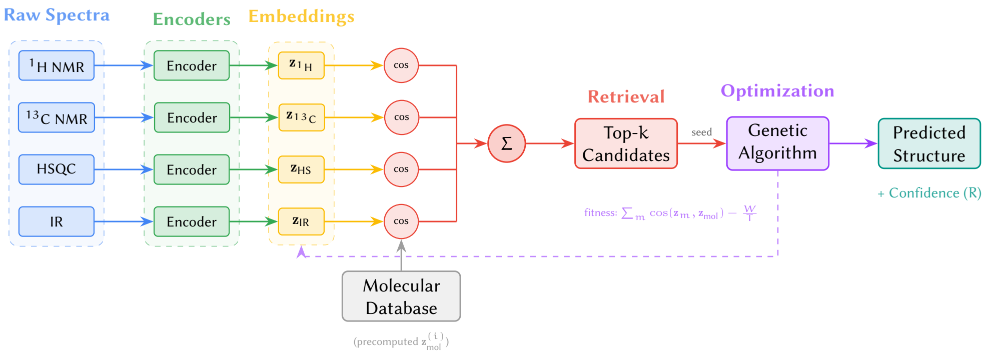

Supplementary Fig. 1: Overview of the SECS structure elucidation pipeline. SECS processes raw experimental spectra without manual preprocessing. **(1) Input:** Any combination of ^{1}H NMR, ^{13}C NMR, HSQC, and IR spectra; missing modalities are handled gracefully. **(2) Encoding:** Modality-specific neural networks (architectures in Supplementary Table 4) map raw spectra to embedding vectors $\mathbf{z}_m$. **(3) Cross-modal retrieval:** Per-modality cosine similarities between spectral embeddings and precomputed molecular embeddings $\mathbf{z}_{mol}^{(i)}$ are summed to rank database candidates, returning the top-k matches. **(4) Optimization:** Retrieved candidates seed a genetic algorithm (GraphGA) that refines structures using summed embedding similarity as the fitness function R. **(5) Output:** Ranked structure predictions with calibrated confidence scores. The dashed feedback loop indicates iterative re-embedding of proposed structures during optimization. This architecture enables database adaptation without retraining—users can substitute the reference database to target different chemical spaces.

Supplementary Note D Contrastive learning

Contrastive learning has been used in chemistry for a variety of tasks.[17–23] In Supplementary Fig. 2, we show how we utilized contrastive learning in our work.

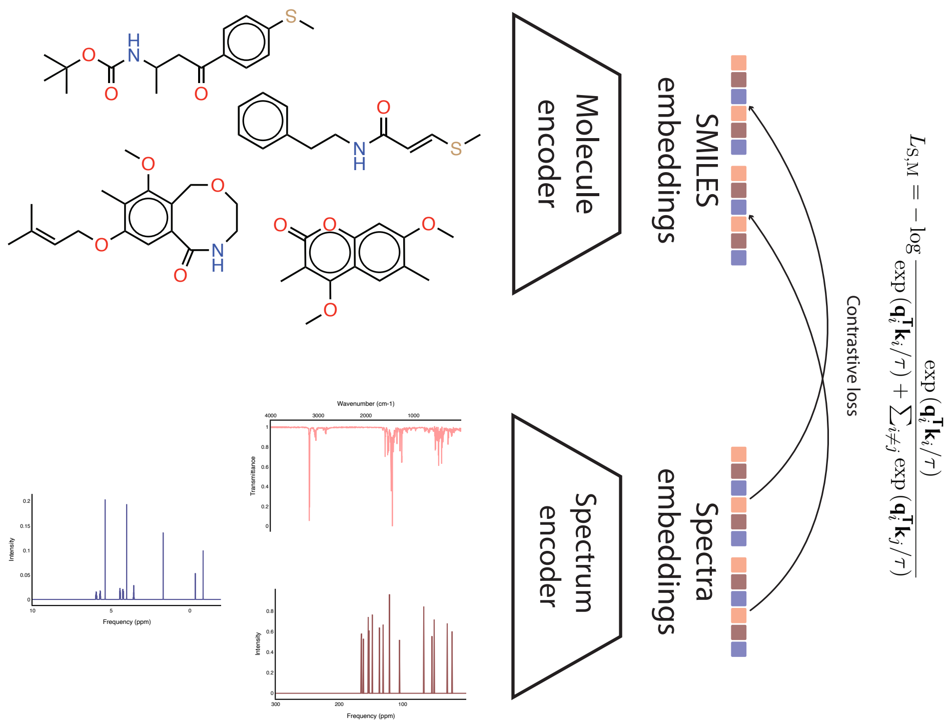

Supplementary Fig. 2: Illustrative example of contrastive learning. Embeddings are generated for SMILES strings and for the spectra represented as vectors. We train four such models: one for the ^{1}H NMR– SMILES pair, one for the ^{13}C NMR–SMILES pair, one for IR– SMILES pair, and the HSQC– SMILES pair. The target of contrastive learning is to align embeddings of various spectra with those of SMILES. The embeddings of spectra and SMILES corresponding to different molecules are set far from each other in the latent space, while the embeddings supposed to represent the same molecule are brought closer to each other in the high-dimensional space.

Supplementary Note E Metrics

Recall@K

$$\text{Recall@K} = \frac{1}{N}\sum_{i=1}^{N} \mathbf{1}[\text{rank}_i \leq K], \tag{1}$$

where N is the total number of queries, K is the cutoff rank (i.e., the number of top predictions considered), rank_i is the rank of the relevant item for query i, and $\mathbf{1}[\cdot]$ is the indicator function. Essentially, it's the fraction of queries where the correct answer appears in the top K results.

Mean Reciprocal Rank (MRR@K)

$$\text{MRR@K} = \frac{1}{N}\sum_{i=1}^{N} \begin{cases} \frac{1}{\text{rank}_i} & \text{if rank}_i \leq K \\ 0 & \text{otherwise} \end{cases} \tag{2}$$

Special Case: K = 1
At $K = 1$, both metrics reduce to accuracy:

$$\text{Recall@1} = \text{MRR@1} = \frac{1}{N}\sum_{i=1}^{N} \mathbf{1}[\text{rank}_i = 1] \tag{3}$$

Fraction Elucidated (Top-K) The fraction of molecules for which the correct structure is identified within the top K predictions of the full pipeline (retrieval + genetic algorithm):

$$\text{Fraction Elucidated@K} = \frac{1}{N}\sum_{i=1}^{N} \mathbf{1}[\text{rank}_i \leq K] \tag{4}$$

where N is the total number of test molecules and rank_i is the rank of the correct structure in the final sorted candidate list for molecule i. This metric differs from retrieval recall in that it evaluates the complete SECS pipeline output, which includes both retrieved database compounds and novel structures proposed by the genetic algorithm.

Multimodal Reward Function (R) The reward function used to score candidates in the genetic algorithm:

$$R = \frac{1}{M}\sum_{x\in\mathcal{S}} d_{\cos}(\varepsilon_x, \varepsilon_{\text{SMILES}}) - \frac{W}{T} \tag{5}$$

where $\mathcal{S} \subseteq \{\mathrm{IR}, {}^{13}\mathrm{C}, {}^{1}\mathrm{H}, \mathrm{HSQC}\}$ is the set of available spectroscopic modalities, $M = |\mathcal{S}|$ is the number of modalities used, $d_{\cos}$ is cosine similarity, ε_x and $\varepsilon_{\mathrm{SMILES}}$ are the embeddings for spectrum x and the molecular SMILES respectively, W is the number of wrong atoms in the proposed molecule, and T is the total number of atoms in the correct molecular formula.

R can also be used as a measure of how calibrated the system is with respect to the total fraction of elucidated structures (*calibrated uncertainty*), which we also show in Fig. 3C. To assess calibration, predictions are grouped into quantile bins based on their reward scores R. For each bin b, the empirical accuracy is computed as:

$$\mathrm{Accuracy}(b) = \frac{1}{|b|} \sum_{i \in b} \mathbf{1}[\mathrm{rank}_i = 1] \tag{6}$$

where $|b|$ is the number of samples in bin b. A well-calibrated system exhibits a linear relationship between the mean reward score of each bin and its corresponding fraction of correctly elucidated spectra, such that higher confidence scores reliably indicate higher accuracy.

Supplementary Note F Retrieval metrics

For computing the retrieval metrics during training, we use the `torchmetrics` library via the mean retrieval ranking (`RetrievalMRR`) and retrieval recall (`RetrievalRecall`) functions.[24] These functions are computed for the aggregated batches in a multi-GPU configuration.

Large scale retrieval For each trained model we computed the performance via the `faiss` vector database (see Supplementary Table 3). We modify the default parameters for a higher accuracy. The wall time with the chosen parameters is higher than the default wall time but leads to increased accuracy of recall.

Supplementary Table 3: `faiss` chosen parameters for the retrieval performance metrics calculation.

	M	construction-ef	search-ef
**Chosen**	32	200	128

We created a vector database for a test set of 38,783 molecules and checked the final performance of a given architecture on this test set. At the same time, for initial development, we relied on the batch metrics described above. In Supplementary Fig. 3, we showcase these metrics for the final model architectures.

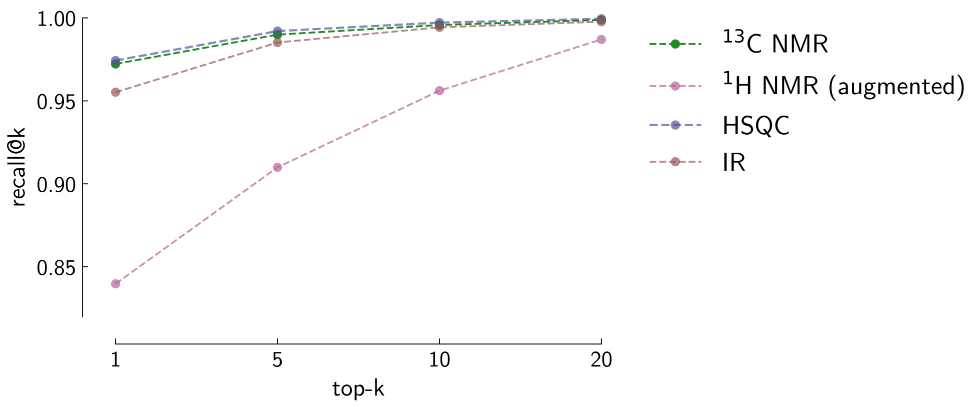

Supplementary Fig. 3: Retrieval recall performance depending on the chosen top-k for different modalities.

Supplementary Note G ^{1}H NMR augmentation

The ^{1}H NMR augmentation serves the purpose of transforming simulation data with idealized peaks, and lack of noise, distortion, and other artifacts typically observed in experiments, into more realistic spectra. In Supplementary Fig. 4 we provide 4 different examples of augmented spectra, with the following subsections describing the augmentation algorithm in detail.

Supplementary Note G.1 Overview

The `augment` function is a comprehensive data augmentation tool designed to enhance ^{1}H NMR spectral datasets by introducing realistic variations and artifacts commonly observed in experimental NMR spectroscopy. This function transforms idealized or simplified NMR spectra into more realistic representations that better reflect the complexity and variability of actual experimental data. An overview of the pipeline is shown in Supplementary Fig. 5.

Supplementary Note G.1.1 Core functionality

The augmentation process begins with peak detection using the `find_peaks` algorithm from the `scipy.signal` module, identifying peaks based on configurable prominence, height, and distance thresholds. The function then applies a series of transformations to simulate various physical and instrumental effects.

Supplementary Note G.2 Peak shape modeling

Each peak is modeled using a pseudo-Voigt profile, combining Gaussian components (representing magnetic field inhomogeneity) and Lorentzian components (representing T_2 relaxation effects). The pseudo-Voigt function is defined as:

$$V(x) = \eta L(x) + (1 - \eta)G(x) \tag{7}$$

where $\eta \in [0.1, 0.9]$ is the mixing parameter, $L(x)$ is the Lorentzian component, and $G(x)$ is the Gaussian component. Gaussian and Lorentzian components are assigned random widths within physically realistic ranges (0.001-0.008 ppm). Peak heights are randomly varied by up to 20% to simulate integration and shimming variations.

Supplementary Note G.3 J-Coupling simulation

With 35% probability, the peaks undergo a *J*-coupling simulation using Pascal's triangle intensity distributions. J-values are randomly selected from 1.0–18.0 Hz, representing typical 1H-1H coupling ranges. Coupling patterns range from doublets

to septets (n+1 rule with up to 6 coupling partners). The splitting pattern for a peak coupled to n equivalent protons follows:

$$\text{Multiplicity} = n + 1 \tag{8}$$

with relative intensities given by the n-th row of Pascal's triangle.

Supplementary Note G.4 Chemical shift perturbations

Spectra undergo random frequency shifts (± 0.10 ppm) to simulate referencing variations. Individual peaks experience small random shifts (± 0.02 ppm) to account for solvent and temperature effects.

Supplementary Note G.5 Spectroscopic artifacts

With 40% probability, ^{13}C satellite peaks are added with realistic 1J(C-H) coupling constants (115-160 Hz) and 1.1% total intensity. Common NMR solvent peaks (CDCl3, DMSO-d6) are incorporated with 60% probability, using appropriate chemical shifts and linewidths. Impurity signals are added with 25% probability, featuring 1-3 impurity peaks with intensities up to 5% of the main spectrum.

Supplementary Note G.6 Phase errors

Phase errors are modeled using both zero-order and first-order corrections. Random phase errors up to ± 15 degrees affect the entire spectrum. Linear phase gradients up to ± 2 degrees per ppm create frequency-dependent phase distortions. Phase errors introduce realistic absorptive/dispersive peak mixtures using $\tan(\phi)$ relationships. The total phase at chemical shift δ is given by:

$$\phi(\delta) = \phi_0 + \phi_1 \cdot (\delta - \delta_{\text{pivot}}) \tag{9}$$

where ϕ_0 is the zero-order phase, ϕ_1 is the first-order phase coefficient, and δ_{pivot} is the pivot frequency.

Supplementary Note G.7 Baseline and noise effects

Random noise addition is scaled to 0.01% of maximum intensity. Polynomial baseline drift of 2nd–5th order is applied with amplitudes up to 0.3% of peak intensity. Final Gaussian broadening (0.0003–0.0015 ppm) simulates additional instrumental effects. The baseline drift is modeled as:

$$B(x) = \sum_{i=0}^{n} a_i x^i \tag{10}$$

where $n \in [2, 5]$ and a_i are random coefficients drawn from a normal distribution.

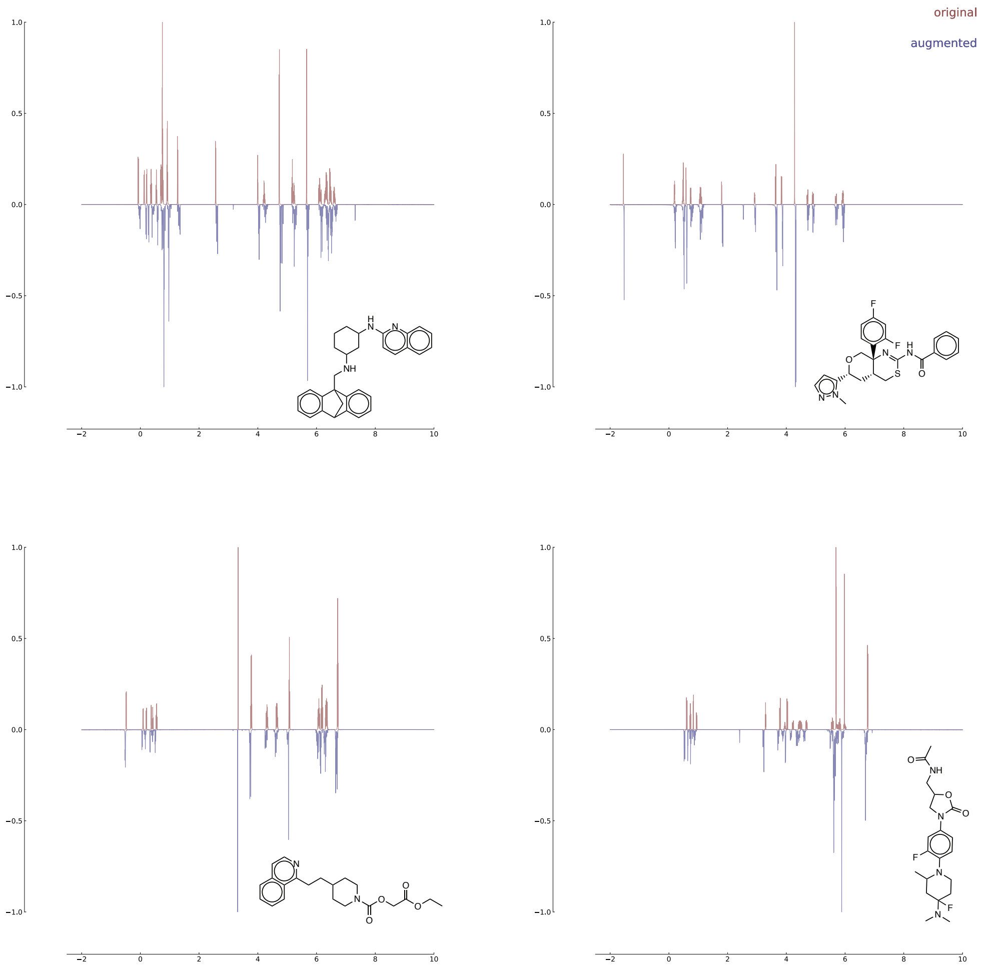

Supplementary Fig. 4: ^{1}H NMR augmentation examples. Here we show four different molecules and their spectra, with the inverted spectra representing the original spectrum augmented using the functions described in Appendix Supplementary Note G.

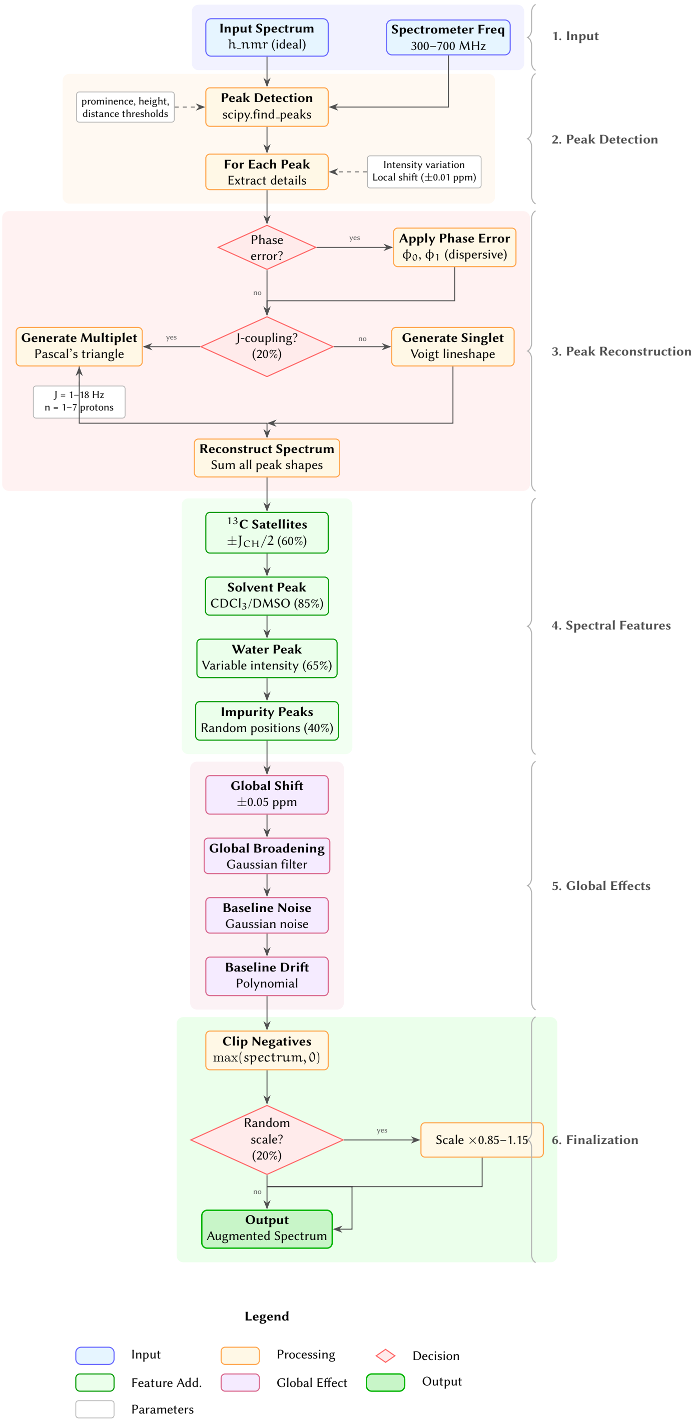

Supplementary Fig. 5: Schematic overview of the augmentation pipeline.

Supplementary Note H Encoder training details

We used the `hydra` package for configuration management and `PyTorch Lightning` for training the model. We made use of the more efficient mixed-precision (`bfloat16` tensors) compared to the standard full precision. In the following paragraphs, we describe in detail the architecture of the spectra encoders, with a summary table of the architectures, parameter counts and the size of latent dimension for each encoder included in Supplementary Table 4.

Supplementary Table 4: Parameter count and architecture summary for the different encoders.

Encoder	Architecture	Parameter count	Latent dim.
SMILES	Transformer	44.4 M	768
IR	standard CNN	7.15 M	512
^{13}C NMR	multi-layer perceptron (MLP)	0.53 M	256
^{1}H NMR	1D ResNet-style CNN + self-attention	86.8 M	1024
HSQC	2D ResNet-style CNN + self-attention	57.8 M	1024

Supplementary Note H.1 ^{1}H NMR encoder architecture

The described model, in its “xlarge” configuration (detailed implementation on GitHub), is a deep one-dimensional convolutional neural network designed to transform an input sequence $\mathbf{X} \in \mathbb{R}^{L \times C_{in}}$ (where L is sequence length, typically 10^4, and C_{in} is the number of input channels, typically 1) into a compressed latent representation $\mathbf{z} \in \mathbb{R}^{D_L}$, with $D_L = 1024$. A simplistic representation of the data flow in the described architecture can be seen in Supplementary Fig. 6.

Supplementary Note H.2 HSQC encoder architecture

The architecture of the HSQC encoder is similar to the one of the ^{1}H NMR encoder. However, it is modified to process 2D inputs. A detailed implementation can be found on GitHub.

Supplementary Note H.3 IR encoder architecture

For IR we use a 1D-CNN, which is used across domains[25–27] to process signals that can be effectively represented as vectors. Here, we have multiple convolutional layers. The first convolutional layer captures the local environments, while the second is supposed to capture long-range interactions between the shifts. The last two convolutional layers are intended to enrich the feature space by providing higher

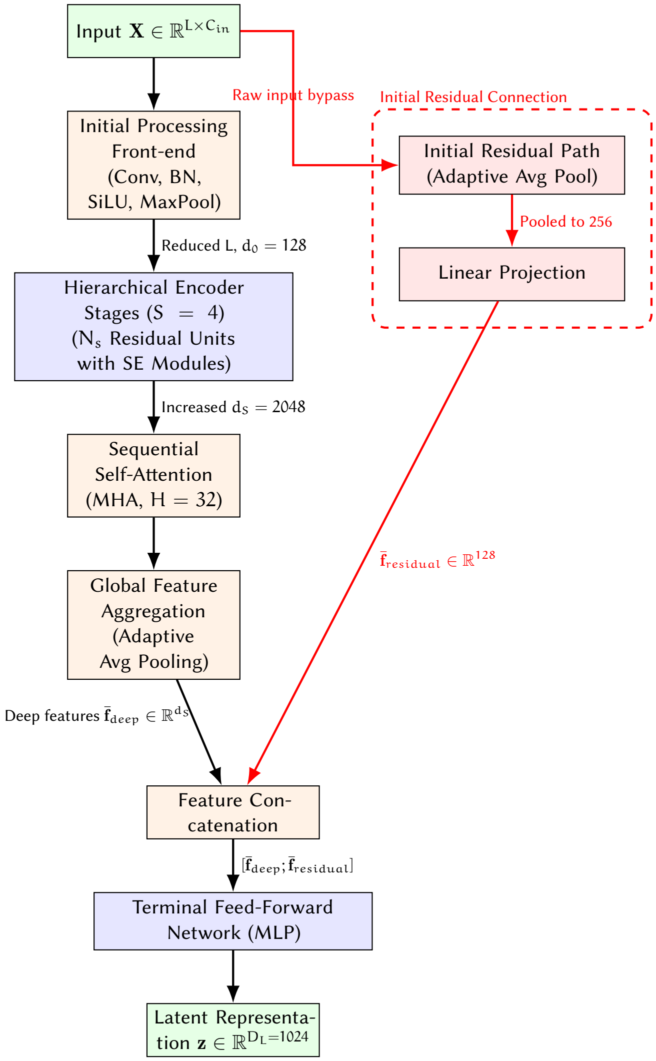

Supplementary Fig. 6: Overview of the ^{1}H NMR Encoder Architecture with Initial Residual Connection. The model processes input through two parallel paths: (1) the main CNN path with front-end processing, hierarchical encoder stages, multi-head self-attention, and global pooling; (2) an initial residual path that directly processes the raw input through adaptive pooling and linear projection. Features from both paths are concatenated before the final feed-forward network projects to the latent space. The residual connection (shown in red) helps preserve low-level input information that might be lost in the deep CNN layers.

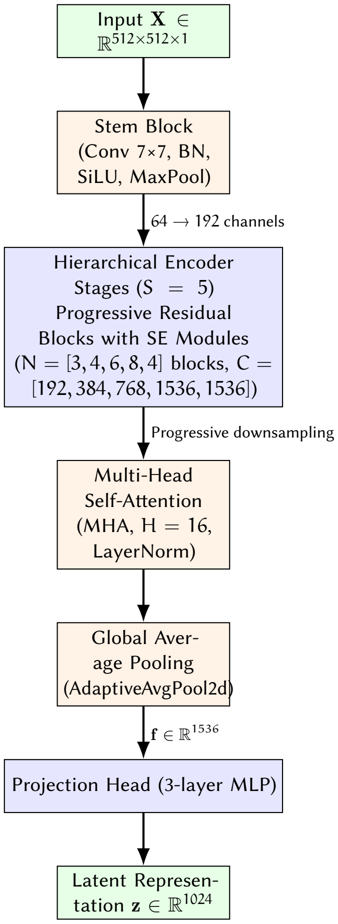

Supplementary Fig. 7: Overview of the HSQC Encoder Architecture. The model processes a 2D input image through a stem block (2D convolution, Batch Normalization, SiLU, and Max Pooling), followed by five hierarchical encoder stages built from residual blocks with Squeeze-and-Excitation modules. A Multi-Head Self-Attention (MHA) layer captures global spatial dependencies, and Adaptive Average Pooling aggregates features before a final Feed-Forward Network (MLP) projects to the latent space.

levels of abstraction.[27–29] We use a sigmoid activation function in the final layer.[30] The full architecture is shown in Supplementary Fig. 8. The hyperparameters are partly inspired by Chen *et al.*[26], who applied a 1D-CNN to near-infrared spectroscopy (NIR). The full architecture is shown in Supplementary Fig. 8.

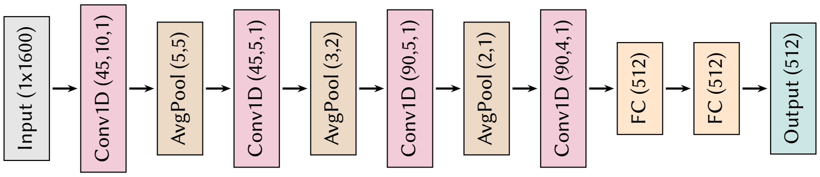

Supplementary Fig. 8: Architecture of the IR encoder. Conv1D - 1D convolution, where the terms in brackets are (number of channels out, kernel dimension, stride). For `AvgPool` the terms in the brackets are (kernel dimension, stride). A dilation of 1 is used for both convolutional and pooling layers.

Supplementary Note H.4 ^{13}C NMR encoder architecture

For encoding ^{13}C NMR, we use a simple architecture — a three-layer feed-forward neural network (FFNN) with layers of sizes $[512, 256, 256]$, each followed by a LeakyReLU activation function. The FFNN is preceded by a `LayerNorm` normalization.

Supplementary Note I Heavy atom distribution

We chose the heavy atom distribution to resemble a similar distribution to Alberts *et al.*[10], averaging a Bertz complexity of 716.

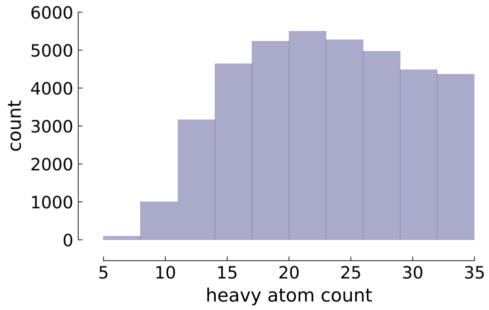

Supplementary Fig. 9: Heavy atom count distribution for the molecules in the simulated test dataset. We observe that beyond 10 heavy atoms, the distribution of heavy atoms is more or less uniform, allowing us to fairly evaluate the model, and identify trends.

Supplementary Note J Hyperparameters for the genetic algorithm

We use an open-source implementation of GraphGA[31–33]. We used a total population size of 20000 (to allow the genetic algorithm to sample from a more diverse set of candidates), an offspring size of 2048, best fraction of the population used for the crossover operations of 0.3. We modified the batched reward calculations to run them on a GPU. The full implementation can be found on GitHub.

Below we show several tables that indicate the detailed settings of the `mol_ga` package related to the various mutation operators. See the following tables: Supplementary Tables 5 to 11.

Supplementary Table 5: Mutation operator selection probabilities in the genetic algorithm. Each mutation type is selected with the indicated probability during the mutation step.

Mutation Operator	Probability
Insert atom	0.15
Change bond order	0.14
Delete cyclic bond	0.14
Add ring	0.14
Delete atom	0.14
Change atom	0.14
Append atom	0.15

Supplementary Table 6: Delete atom SMARTS reaction probabilities. Reactions are selected based on the degree (D) of the atom to be deleted.

Target Atom Degree	SMARTS Pattern	Probability
D1 (terminal)	`[*:1]~[D1:2]>>[*:1]`	0.250
D2	`[*:1]~[D2:2]~[*:3]>>[*:1]-[*:3]`	0.250
D3	`[*:1]~[D3:2](...)~[*:4]>>...`	0.250
D4 (variant 1)	`[*:1]~[D4:2](...)(...)>>...`	0.1875
D4 (variant 2)	`[*:1]~[D4:2](...)(...)>>...`	0.0625

Supplementary Table 7: Append atom probabilities. An atom is appended to the molecule with the specified bond order and element distribution.

Bond Order	Available Elements	Probability
Single	C, N, O, F, S, Cl, Br (uniform)	0.60
Double	C, N, O (uniform)	0.35
Triple	C, N (uniform)	0.05

Supplementary Table 8: Insert atom probabilities. An atom is inserted between two existing atoms with the specified bond order and element distribution.

Bond Order	Available Elements	Probability
Single	C, N, O, S (uniform)	0.60
Double	C, N (uniform)	0.35
Triple	C (uniform)	0.05

Supplementary Table 9: Change atom type probabilities. An existing atom is transmuted to a different element with uniform probability among eligible elements.

Element	Atomic Number	Probability
C	6	0.15
N	7	0.15
O	8	0.14
F	9	0.14
S	16	0.14
Cl	17	0.14
Br	35	0.14

Supplementary Table 10: Change bond order probabilities in the genetic algorithm.

Transformation	Probability
Non-single $\rightarrow$ single	0.45
Single $\rightarrow$ double	0.45
Triple $\rightarrow$ double	0.05
Non-triple $\rightarrow$ triple	0.05

Supplementary Table 11: Ring formation probabilities in the genetic algorithm.

Ring Size	Probability
3-membered	0.05
4-membered	0.05
5-membered	0.45
6-membered	0.45

Supplementary Note J.1 The relationship between the number of generations and the reward

In Supplementary Table 12, we present the relationship between the number of generations and the max, mean scores and the respective standard deviations. We do observe that the GA most frequently converges within the first few generations.

Supplementary Table 12: Tabulated scores per generation. The max and mean score across generations 1 to 15, along with their respective standard deviation, with the final population of 10000 molecules.

Generation	Mean score	Max score
1	0.027 ± 0.098	0.777 ± 0.122
2	0.027 ± 0.092	0.813 ± 0.112
3	0.030 ± 0.089	0.833 ± 0.102
4	0.034 ± 0.088	0.846 ± 0.093
5	0.039 ± 0.088	0.854 ± 0.086
6	0.043 ± 0.088	0.861 ± 0.081
7	0.065 ± 0.093	0.865 ± 0.077
8	0.098 ± 0.097	0.868 ± 0.075
9	0.125 ± 0.099	0.871 ± 0.071
10	0.149 ± 0.101	0.874 ± 0.068
11	0.172 ± 0.103	0.876 ± 0.065
12	0.194 ± 0.105	0.878 ± 0.064
13	0.215 ± 0.107	0.879 ± 0.062
14	0.234 ± 0.109	0.880 ± 0.061
15	0.253 ± 0.111	0.881 ± 0.060

Supplementary Note K Ablations

Supplementary Note K.1 Ablating the effect of including multiple molecular formulas

We analyzed the effect of building the initial population with one vs. multiple molecular formula. The molecular formula are built based on the number of the different atoms present in it: e.g., using a slightly different number of carbon atoms, of hydrogen atoms or atoms from any other elements. This creates a list of different molecular formula that are then used to filter the database.

According to Supplementary Fig. 10, the GA returns better results when the initial population is drawn from a more diverse set of formulas.

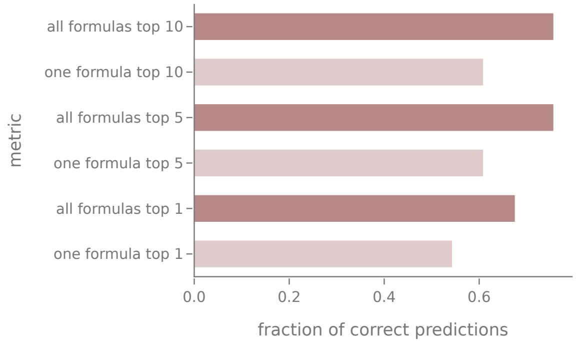

Supplementary Fig. 10: Comparative performance of the SECS pipeline when the initial population is drawn from a pool of molecules with slightly varying molecular formulas vs. using just the molecular formula of the correct molecule. (based on 100 examples from the test set) Only one random seed has been used for this ablation.

Supplementary Note K.2 Additional investigation of the benefit of adding new modalities

In this section, we provide additional evidence for the benefit of adding new modalities, besides the simpler metric of ranking within top-N candidates. From Fig. 3A, we see a boost in performance with every added modality, but the exact rank of each correct prediction is also an important aspect.

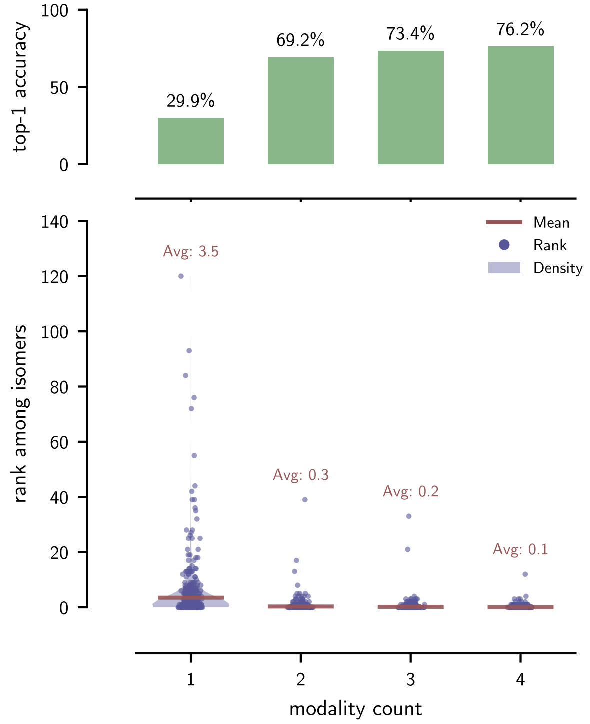

Supplementary Fig. 11: The effect of incrementally adding new modalities. The rank subplot follows the same order of addition as in Fig. 3B in the main text). We observe that the addition of new modalities limits the amount of extreme ranks of the correct prediction, always being below 20 for all four modalities, when the correct molecule is found, indicating that our system becomes increasingly confident.

Supplementary Note K.3 Ablating the effect of augmentations and resolutions

We test different levels of augmentation (for all probabilities mentioned in this section set at the same level) and observe the retrieval performance within the 2369 experimental spectra available to us. From Supplementary Fig. 12a, we can conclude that very high augmentation levels and no augmentation at all have little correlation with experimental data, with no augmentation performing the worst. Using custom levels (as those described in Supplementary Note G) for each separate type of augmentation results in the best performance.

In the context of ^1H NMR we also evaluate the impact of the resolutions of the spectra on the recall within a large test set (38.8k) changes. It can be observed in Supplementary Fig. 12b that the resolution of the spectrum has no great impact on the performance. However, if a high-resolution model is tested on low-resolution spectra, the performance drops. The same is true when testing low-resolution models on high-resolution spectra. For this reason, we also train our final models on different levels of resolution to be robust to resolution changes (ranging from a resolution of 500 to 10000). The resolution is changed by performing window averaging.

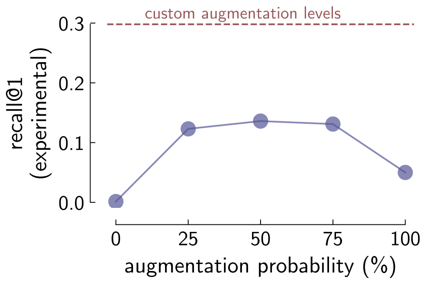

(a) Impact of different augmentation levels on experimental data retrieval performance (within a set of 2.37k spectra-molecule pairs). The best performance is achieved when custom augmentation levels are chosen.

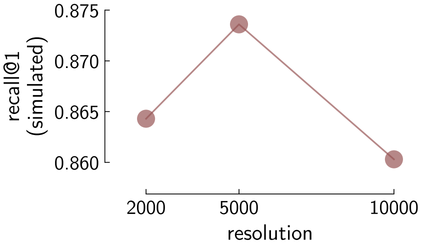

(b) Impact of different spectra resolutions on the recall@1 among simulated spectra. The models are trained for five epochs and tested on the 38.8k test set of simulated spectra. The resolution has no effect on the overall recall.

Supplementary Fig. 12: Ablating the effects of augmentation on the experimental data, and the effect of the ^1H NMR resolution on the simulated test set retrieval.

Supplementary Note L Domain of applicability

We analyzed several parameters, such as the maximum Tanimoto similarity of any molecule from the initial population to the target molecule, the maximum cosine similarity between the spectra embedding and any compound (for the purpose of the experiments the correct molecule is removed) in the initial population to the correct molecule, and the Bertz complexity.[34,35] The results of these analyses are shown in Supplementary Fig. 13. The performance of our algorithm directly depends on all of these factors. For instance, the distribution of Bertz complexities is skewed towards higher values when the correct molecule is not found. The maximum Tanimoto similarity of the compounds in the initial population also plays a pivotal role in determining whether the right compound is found. The maximum initial Tanimoto similarities are skewed towards the range $0.8 - 1$.

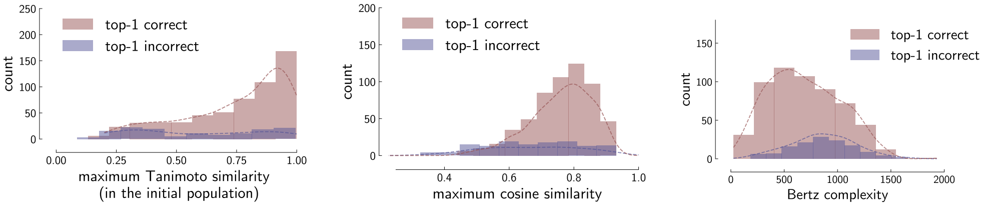

(a) Tanimoto similarity **(b)** cosine similarity **(c)** Bertz CT

Supplementary Fig. 13: The distribution of correct vs incorrect predictions (recall@1=True or recall@1=False). The metrics used to understand the domain of applicability are (a) Tanimoto similarity, (b) cosine similarity, and (c) Bertz CT of the correct molecule. The dataset used for this analysis is the same test set used to create Fig. 2 in the main text with the performance for three different seeds and four spectra (IR, ^{1}H NMR, ^{13}C NMR, HSQC).

Supplementary Note M Comparison of experimental and simulated training sets

Comparing the experimental dataset (training and test sets) with the simulated data provided by IBM, we observe that the experimental data covers a much smaller part of the chemical space.

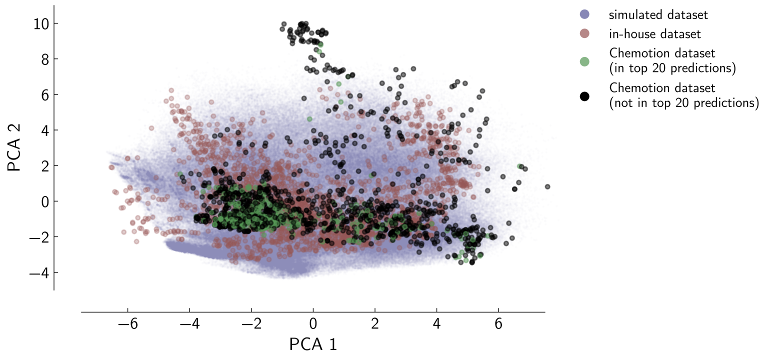

Supplementary Fig. 14: Chemical space comparison betweeen the simulated dataset, the in-house experimental dataset and the mined Chemotion dataset. The principal component analysis (PCA) has been performed on the Morgan fingerprint of the molecules. We color different subsets of the Chemotion dataset based on whether the correct molecule is in the top-20 results provided by `SECS` (green for being found, and black for not).

We also plot the distribution of the molecular weights for three datasets: the simulated IBM dataset (training set), the in-house experimental training data, and the data mined from the Chemotion repository. The results are displayed in Supplementary Fig. 15.

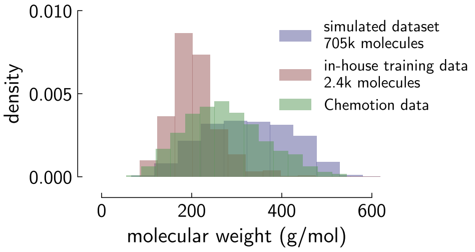

Supplementary Fig. 15: Molecular weight distribution of the experimental data vs the simulated data. There are two experimental datasets: the Chemotion dataset and our in-house collected dataset. The Chemotion data more closely matches the distribution of the simulated data.

Supplementary Note N Comparison with human performance

To contextualize the performance of SECS, we selected 20 entries of the Chemotion dataset for a study with expert chemists.

For the study, we developed a web interface using the visualizer framework.[36]

On first use, participants are shown instructions and a survey about their experience in structure elucidation. After that, they are shown a list of 20 examples they can solve. We track the time starting with them opening the first example. If participants do not move their mouse for more than 60 s the timer is stopped and resumed once the mouse is moved again.

Participants always see a ^{1}H-NMR spectrum in the NMR processing tool NMRium,[37] which also provides utilities for (automatic) integration and peak picking. Participants are also allowed to use any tool of their choice. In addition, we also always show the double bond equivalents as well as the molecular mass of the true molecule and their current proposal, which they can draw in a molecule editor.[38] For half of the compounds, we also show a list of the top-20 proposals of SECS.

Interestingly, we obtain mixed results when it comes to the human-AI collaboration. Some participants performed better when using AI, some performed the same, and some had worse results. The human-AI collaboration component is still an active field of research, and mixed results have been obtained across domains.[39,40]

The human challenge view is available at `https://www.cheminfo.org/?viewURL=https%3A%2F%2Fcouch.cheminfo.org%2Fcheminfo-public%2F5ef29a5ce78c2a23470390a551af445b%2Fview.json&loadversion=true&fillsearch=Kevin+Challenge&key=5150c2150229a291bd4cc9609c4b15fe&folder=60bc18ef-46dd-4fd8-ad2e-64b6bbc2de23&server=https%3A%2F%2Fjsmol.cheminfo.org&topic=nmrium2025` (see Supplementary Fig. 16 for a screenshot of the app).

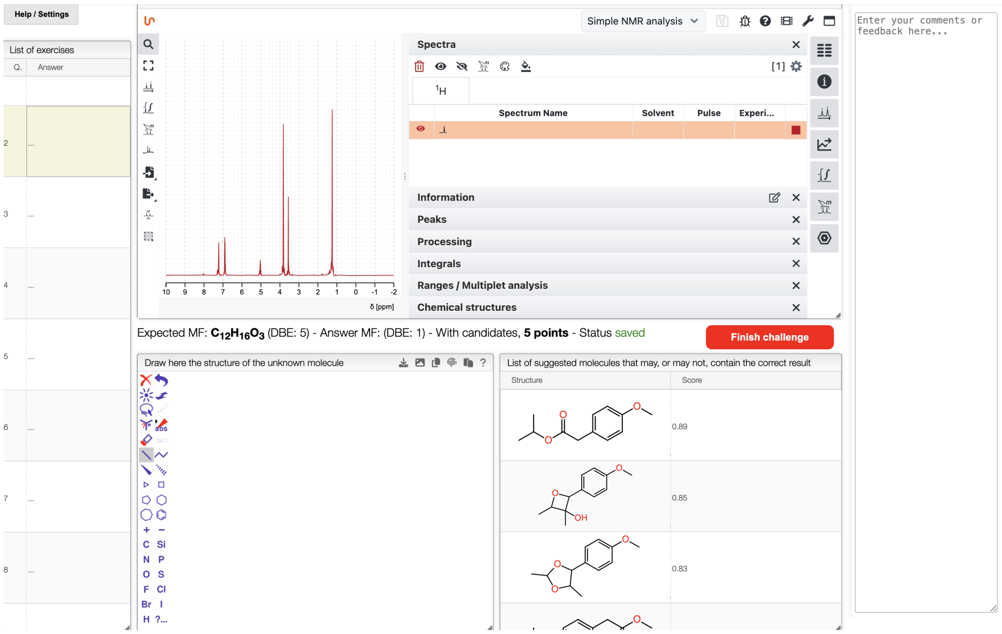

Supplementary Fig. 16: Screenshot of the web app used for testing the performance of humans. Each person was shown only the spectrum (via NMRium[41]) and its molecular formula, with a box for drawing the final proposal. A box with the proposals produced by SECS is filled half of the time (like in the example above).

Supplementary Note O Web interfaces

We developed multiple web interfaces for this study. All have been developed in the visualizer framework.

Data visualization `https://www.cheminfo.org/?viewURL=https://couch.cheminfo.org/cheminfo-public/dd914af4faab9c30bef3da09f208d74d/view.json` can be used to visualize the data. A screenshot of the visualization app is available in Supplementary Fig. 17.

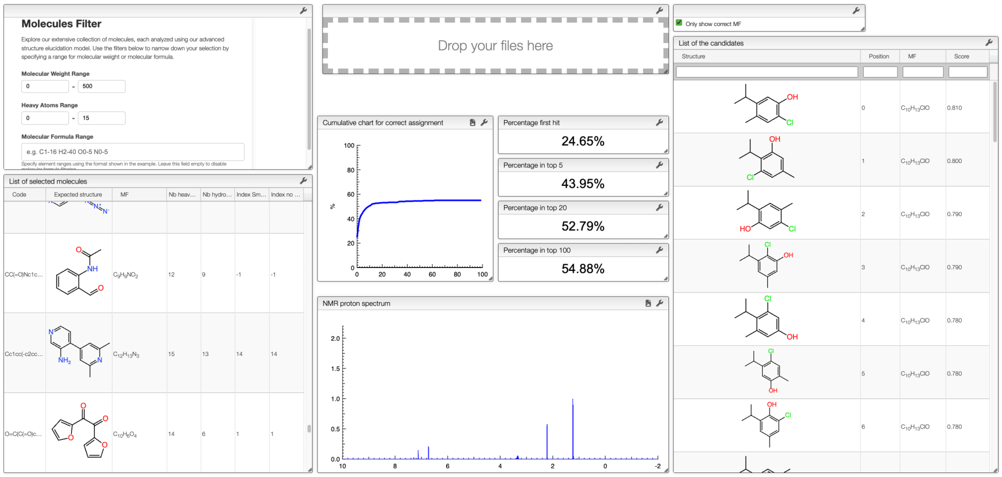

Supplementary Fig. 17: Screenshot of the data visualization app. The app contains four section: the molecule filter (by molecular formula, molecular weight or heavy atom count), the top-k vs performance curve, the ranked list of predictions for each input and the display of the raw data (spectra and molecules).

SECS app We also provide an app for testing SECS. The backend is implemented as RESTful interfaces using FastAPI. In our architecture, one service manages the vector database. The other handles the main application logic.

The front-end is implemented using the visualizer framework.

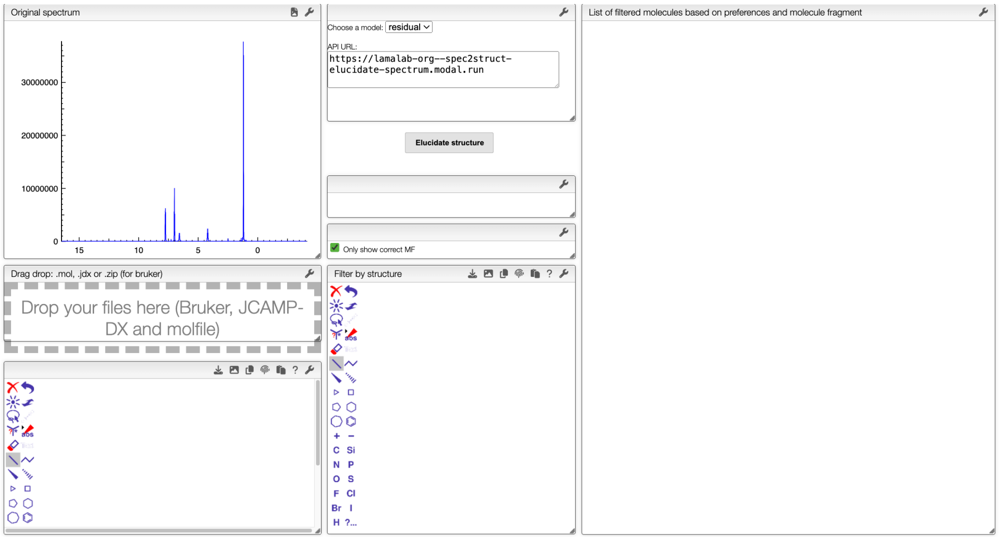

Supplementary Fig. 18: Screenshot of the web app that can be used to test SECS. A Bruker or JCAMP-DX file coming directly from the ^{1}H NMR spectrometer can be dragged and dropped. After some time the first results will appear (2–4 minutes) as a ranked list. One can filter the list by some substructure that can be drawn directly in the box.

The app is available at `https://secs.lamalab.org`. A screenshot of the visualization app is available in Supplementary Fig. 18.

References

1. Howarth, A., Ermanis, K. & Goodman, J. M. DP4-AI automated NMR data analysis: straight from spectrometer to structure. *Chemical science* **11,** 4351–4359 (2020).
2. Enders, A. A., North, N. M., Fensore, C. M., Velez-Alvarez, J. & Allen, H. C. Functional group identification for FTIR spectra using image-based machine learning models. *Analytical Chemistry* **93,** 9711–9718 (2021).
3. Lee, G., Shim, H., Cho, J. & Choi, S.-I. Machine-Learning Approach to Identify Organic Functional Groups from FT-IR and NMR Spectral Data. *ACS omega* **10,** 12717–12723 (2025).
4. Alberts, M., Hartrampf, N. & Laino, T. From Spectra to Structure: AI-Powered 31P NMR Interpretation. *Analytical Chemistry* **97,** 15736–15742 (2025).
5. Sridharan, B., Mehta, S., Pathak, Y. & Priyakumar, U. D. Deep reinforcement learning for molecular inverse problem of nuclear magnetic resonance spectra to molecular structure. *The Journal of Physical Chemistry Letters* **13,** 4924–4933 (2022).
6. Alberts, M., Zipoli, F. & Laino, T. Setting new benchmarks in AI-driven infrared structure elucidation. *Digital Discovery* (2025).
7. Alberts, M., Laino, T. & Vaucher, A. C. Leveraging infrared spectroscopy for automated structure elucidation. *Communications Chemistry* **7,** 268 (2024).
8. Kanakala, G. C., Sridharan, B. & Priyakumar, U. D. Spectra to structure: contrastive learning framework for library ranking and generating molecular structures for infrared spectra. *Digital Discovery* **3,** 2417–2423 (2024).
9. Wu, W., Leonardis, A., Jiao, J., Jiang, J. & Chen, L. Transformer-Based Models for Predicting Molecular Structures from Infrared Spectra Using Patch-Based Self-Attention. *The Journal of Physical Chemistry A* **129,** 2077–2085 (2025).
10. Alberts, M., Zipoli, F. & Vaucher, A. C. Learning the Language of NMR: Structure Elucidation from NMR spectra using Transformer Models. *ChemRxiv* (2023).
11. Pesek, M. *et al.* Database independent automated structure elucidation of organic molecules based on IR, 1H NMR, 13C NMR, and MS data. *Journal of chemical information and modeling* **61,** 756–763 (2020).
12. Devata, S. *et al.* DeepSPInN – deep reinforcement learning for molecular structure prediction from infrared and 13C NMR spectra. en. *Digital Discovery.* Publisher: RSC. (2024) (Mar. 2024).
13. Wenk, M., Nuzillard, J.-M. & Steinbeck, C. Sherlock—a free and open-source system for the computer-assisted structure elucidation of organic compounds from NMR data. *Molecules* **28,** 1448 (2023).

14. Hu, F., Chen, M. S., Rotskoff, G. M., Kanan, M. W. & Markland, T. E. Accurate and efficient structure elucidation from routine one-dimensional NMR spectra using multitask machine learning. *ACS Central Science* (2024).

15. Priessner, M. *et al.* Enhancing Molecular Structure Elucidation: MultiModalTransformer for both simulated and experimental spectra. *ChemRxiv 10.26434/chemrxiv-2024-zmmnw* (Nov. 2024).

16. Alberts, M., Schilter, O., Zipoli, F., Hartrampf, N. & Laino, T. Unraveling Molecular Structure: A Multimodal Spectroscopic Dataset for Chemistry. *arXiv preprint arXiv: 2407.17492* (2024).

17. Seidl, P., Vall, A., Hochreiter, S. & Klambauer, G. *Enhancing activity prediction models in drug discovery with the ability to understand human language* in *International Conference on Machine Learning* (2023), 30458–30490.

18. Sanchez-Fernandez, A., Rumetshofer, E., Hochreiter, S. & Klambauer, G. CLOOME: contrastive learning unlocks bioimaging databases for queries with chemical structures. *Nature Communications* **14,** 7339 (2023).

19. Yang, Z. *et al.* Cross-modal retrieval between 13C NMR spectra and structures for compound identification using deep contrastive learning. *Analytical Chemistry* **93,** 16947–16955 (2021).

20. Liu, S. *et al.* Multi-modal molecule structure–text model for text-based retrieval and editing. *Nature Machine Intelligence* **5,** 1447–1457 (2023).

21. Mirza, A., Starke, S., Merdivan, E. & Jablonka, K. M. *Bridging chemical modalities by aligning embeddings* in *AI for Accelerated Materials Design-Vienna 2024* (2024).

22. Mirza, A. et al. *MatBind: Probing the multimodality of materials science with contrastive learning* in *AI for Accelerated Materials Design-ICLR 2025* (2025).

23. Wang, Y., Wang, J., Cao, Z. & Barati Farimani, A. Molecular contrastive learning of representations via graph neural networks. *Nature Machine Intelligence* **4,** 279–287 (2022).

24. Detlefsen, N. S. *et al.* TorchMetrics - Measuring Reproducibility in PyTorch. *Journal of Open Source Software.* Repository: https://github.com/Lightning-AI/torchmetrics (Feb. 2022).

25. Wang, H. *et al.* Rapid identification of X-ray diffraction patterns based on very limited data by interpretable convolutional neural networks. *Journal of chemical information and modeling* **60,** 2004–2011 (2020).

26. Chen, X. *et al.* Probing 1D convolutional neural network adapted to near-infrared spectroscopy for efficient classification of mixed fish. *Spectrochimica Acta Part A: Molecular and Biomolecular Spectroscopy* **279,** 121350 (2022).

27. Litsa, E. E., Chenthamarakshan, V., Das, P. & Kavraki, L. E. An end-to-end deep learning framework for translating mass spectra to de-novo molecules. *Communications Chemistry* **6,** 132 (2023).
28. Krizhevsky, A., Sutskever, I. & Hinton, G. E. Imagenet classification with deep convolutional neural networks. *Advances in neural information processing systems* **25** (2012).
29. Fang, W. *et al.* Deep residual learning in spiking neural networks. *Advances in Neural Information Processing Systems* **34,** 21056–21069 (2021).
30. Ige, A. O. & Sibiya, M. State-of-the-Art in 1D Convolutional Neural Networks: A Survey. *IEEE Access* **12,** 144082–144105 (2024).
31. Brown, N., Fiscato, M., Segler, M. H. & Vaucher, A. C. GuacaMol: benchmarking models for de novo molecular design. *Journal of chemical information and modeling* **59,** 1096–1108 (2019).
32. Jensen, J. H. A graph-based genetic algorithm and generative model/Monte Carlo tree search for the exploration of chemical space. *Chemical science* **10,** 3567–3572 (2019).
33. Tripp, A. & Hernández-Lobato, J. M. Genetic algorithms are strong baselines for molecule generation. *arXiv preprint arXiv:2310.09267* (2023).
34. Tanimoto, T. T. Elementary mathematical theory of classification and prediction (1958).
35. Bertz, S. H. The first general index of molecular complexity. *Journal of the American Chemical Society* **103,** 3599–3601 (1981).
36. Pellet, N. *Visualizer: A Tool for Rendering Complex Datasets* `https://github.com/NPellet/visualizer`. 2024.
37. Patiny, L. *et al.* NMRium: Teaching nuclear magnetic resonance spectra interpretation in an online platform. *Beilstein Journal of Organic Chemistry* **20,** 25–31 (Jan. 2024).
38. Wahl, J. & Sander, T. Fully Automated Creation of Virtual Chemical Fragment Spaces Using the Open-Source Library OpenChemLib. *Journal of Chemical Information and Modeling* **62,** 2202–2211 (Jan. 2022).
39. Yu, F. *et al.* Heterogeneity and predictors of the effects of AI assistance on radiologists. *Nature Medicine* **30,** 837–849 (2024).
40. Spillias, S. *et al.* Human-AI collaboration to identify literature for evidence synthesis. *Cell Reports Sustainability* **1** (2024).
41. Patiny, L. *et al.* NMRium: Teaching nuclear magnetic resonance spectra interpretation in an online platform. *Beilstein journal of organic chemistry* **20,** 25–31 (2024).
